# Management of Poor Responders in IVF: Is There Anything New?

**DOI:** 10.1155/2014/352098

**Published:** 2014-07-20

**Authors:** Filippo Ubaldi, Alberto Vaiarelli, Rosario D'Anna, Laura Rienzi

**Affiliations:** ^1^GENERA, Centre for Reproductive Medicine, Valle Giulia Clinic, Rome, Italy; ^2^Department of Gynecological/Obstetrical Sciences and Reproductive Medicine, University Hospital “G. Martino,” Messina, Italy

## Abstract

Despite the fact that in the last two decades an enormous number of papers on the topic of poor ovarian response have been published in the literature, so far it has been impossible to identify any efficient treatment to improve the ovarian response and the clinical outcome of this group of patients. The incidence of poor ovarian responders among infertile women has been estimated at 9–24% but according to recent reviews, it seems to have slightly increased. The limitation in quantifying the incidence of these patients among the infertile population is due to the difficulty of a clear definition in literature. A recent paper by the Bologna ESHRE working group on poor ovarian response has been the first real attempt to find a common definition. Current literature proposes new risk factors which could be the cause of a reduction in ovarian reserve, which also includes genetic factors. This represents the first necessary step towards finding applicable solutions for these patients. To date, there is a substantial lack of literature that identifies an ideal protocol for these patients. The use of the “Bologna criteria” and the introduction of long acting gonadotropin in clinical practice have given rise to new promising stimulation protocols for this group of patients.

## 1. Introduction

In the field of assisted reproductive technologies great steps forward have been made in recent years in terms of clinical knowledge and technological development especially in IVF laboratories. One of the fundamental steps to reach the success is still related to the number of eggs obtained after hormonal stimulation by gonadotropins in combination with GnRH analogues. In patients defined “poor responders,” the limited number of obtained eggs remains the main problem in optimizing the live birth rates. In fact, as a result of a lower number of oocytes retrieved, there are fewer embryos to select and transfer and subsequently these patients have lower pregnancy rates per transfer and lower cumulative pregnancy rates per started cycle compared with normal responders. Although the concept of poor ovarian response was introduced over 30 years ago, we had not had a common definition of poor responder patients until 2011. In fact, Polyzos and Devroey [1] emphasized enormous variability of the definitions of poor responder patients proposals from the literature (in 47 randomized trials, 41 different definitions). These results confirm the difficulty in obtaining an exact incidence of this condition (that has been estimated at 9–24% but it seems to be slightly increased in the last decade [1–6]), the incapacity to compare the results of different trials and therefore to identify the best treatment. Recently, the ESHRE working group on poor ovarian response has finally given a common definition of “poor responder,” where at least two of the following three features must be present: (a) advanced maternal age or any other risk factor for poor ovarian response (POR); (b) a previous POR; and (c) an abnormal ovarian reserve test [7]. It is desirable that this classification will be used in the future in the studies dealing with poor ovarian reserve. We can observe a physiological decline of the “follicular heritage” in every woman over time with a marked increase in the rate of follicular disappearance from age 37 to 38 years onwards [8]. From this moment, the time to the menopause takes about 10–13 years. In poor responders the mechanism of ovarian insufficiency is prematurely determined and not fully understood. Some causes of decrease in ovarian reserve have been identified: ovarian surgery especially in case of endometrioma [9–12], genetic defects, chemotherapy, radiotherapy, autoimmune disorders, single ovary, chronic smoking, and unexplained infertility [13, 14]. Moreover, new risk factors of low ovarian response have been proposed: diabetes mellitus Type I [15], transfusion-dependent B-thalassemia [16], and uterine artery embolization for the treatment of uterine leiomyoma [17, 18]. However, in most cases the mechanism involved in follicular depletion is still not clear [13]. It has been suggested that the reduced number of oocytes could be correlated to a reduction of their quality, which is clinically translated into a reduction of the implantation rates and an increase of early pregnancy loss [19]. Conversely, because of the lack of a clear correlation between “quantity” and “quality,” different authors have suggested that poor responders “per se” do not represent a lower chance of success in IVF, with the age of the woman being the most important predictor of live birth rate [20, 21]. However, very large studies have shown that this group of patients has reduced pregnancy rates compared with normal responders independently from the treatment protocol used [22] and from the age of the patient [23, 24]. In this group of difficult patients it is thus clear that to optimize the clinical results in IVF it is not only important to predict the ovarian reserve but also, especially, to tailor the best stimulation protocol to exploit fully the ovarian reserve and optimize the number of oocytes to be retrieved. Only with recent advent of new reliable biomarkers of ovarian reserve better strategies for the management of these patients have been suggested [25].

## 2. Prediction of Ovarian Response

Predicting ovarian response before starting hormonal stimulation is the only way to administer an efficient and safe treatment. The most important predictors of the ovarian response to hormonal stimulation are age, biochemical parameters (basal FSH levels in the early follicular phase, serum antimullerian hormone [AMH]), and morphological characteristics (antral follicular count [AFC] and ovarian volume) [26]. Although ovarian reserve declines with age [8], it does not represent an optimal predictor of ovarian response [25]. Basal serum FSH has been considered for many years as the most important and reliable marker to predict the ovarian response to stimulation in IVF/ICSI cycles. Basal serum FSH concentrations begin to rise on average a decade or more before the menopause [27]. This is caused by the reduction of the negative feedback of the FSH-modulating proteins from the ovary, mainly inhibin-A and inhibin-B [28] secondary to the reduction of early antral follicles [29, 30]. More recently it has been demonstrated that it is a good predictor only at very high threshold levels (>FSH 12 mIU/mL) predicting a very compromised ovarian reserve [26]. AMH is produced from preantral follicles and small antral follicles up to 7-8 mm. It inhibits FSH mediated granulosa cell proliferation, follicular growth, and aromatase activity [31]. AMH provides a quantitative evaluation of the amount of follicles that cannot be assessed by AFC. For this reason AMH level has a very low inter- and intracycle variability remaining stable during menstrual cycles [31, 32] but some factors like smoking and current oral contraceptive pills [33] can determine variability. Moreover, the time interval between serum AMH and the beginning of the ovarian stimulation up to 12 months does not seem to modify the capacity of the marker to identify patients with low ovarian response [34]. A recent meta-analysis [35] has confirmed AMH an excellent predictor of poor ovarian response to ovarian stimulation although the ideal test is the response of the ovaries to ovarian stimulation itself.

However, the same meta-analysis underlines that AMH and AFC, alone or in combination, did not improve the prediction of ongoing pregnancy rate, with the age of the woman being the most important factor related to live birth rates [35]. Lastly, some data underlines the role of body mass index (BMI) in female reproduction: obese poor responders could have a lower pregnancy rate than nonobese poor responders [36, 37].

## 3. Why Is This Topic So Controversial and So Current?

The difficulties encountered by researchers in finding “real” options in the treatment of poor responders are due to the following: (1) many studies are represented by a small number of patients limiting the power to find a significant difference between the various treatments; (2) numerous definitions of “poor responder” reported in the literature has led to the presence of a heterogeneous group of patients; (3) causes and mechanisms leading to poor ovarian reserve are still unclear, especially in young women they are largely unknown; (4) different end points have been used in the studies to evaluate the outcomes of this group of patients; (5) impossibility to compare results from different studies due to the presence of numerous “bias”; and (6) limited value of some meta-analyses ruling out many observational studies.

In terms of what we discussed, reviews and meta-analyses conclude that there is insufficient evidence to recommend the use of particular intervention to improve outcomes in this group of patients.

## 4. Definition of Poor Ovarian Response: The “Bologna Criteria”

Before 2011, a huge variety of definitions for poor ovarian response have been proposed and published in the literature: the number of mature follicles on the day of human chorionic gonadotropin (HCG) administration (<2 to <5) [38–40], the number of oocytes retrieved (<4 to <6) [41, 42], the serum estradiol concentrations (<100 pg/mL on day 5 of stimulation or <300 to <600 pg/mL on the day of HCG) [39, 43, 44], or the total gonadotropin dose used and/or the daily stimulation dose [45–47] and/or prolonged duration of gonadotropin stimulation [48]. In 2011, a panel of experts in reproductive medicine gathered together in an ESHRE Campus on poor responders held in Bologna with the aim to find a common and universal definition of poor ovarian response trying to find simple, clearly defined, and reproducible criteria. The Bologna ESHRE criteria represent the first real attempt by the scientific community to unify the many definitions proposed to identify poor responder patients by establishing a definite point from which to begin and how to find therapeutic strategies. It was concluded that “poor ovarian responders” should be considered patients having at least two of the following criteria: (1) a previous episode of poor ovarian response (≤3 oocytes) with a standard dose of medication; (2) an abnormal ovarian reserve with AFC <5–7 follicles or AMH <0.5–1.1 ng/mL; (3) women above 40 years of age or presenting other risk factors for poor response such as previous ovarian surgery, genetic defects, chemotherapy, radiotherapy, and autoimmune disorders [7]. To identify poor responders among women over the age of 40, it is however sufficient to document a reduced ovarian reserve, in the absence of ovarian stimulation. The aim of the “Bologna criteria” is to select homogeneous populations for future trials where new strategies could be suggested and tested. The “Bologna criteria” although accepted by the vast majority of the scientific community have raised some criticism. A letter to the editor on the ESHRE consensus on the definition of “poor ovarian response” to ovarian stimulation [49] underlines that although the first step has been made with the introduction of “Bologna criteria,” “the work is yet to be accomplished.” In accordance with the literature, guidelines for physicians should be necessary to identify risk factors and to integrate these with the diagnosis of poor ovarian response, especially for young women. Although comments and suggestion reported by Younis could be useful to better identify poor ovarian responders especially in young women, this is the only paper so far that questions the ESHRE consensus paper.

## 5. Is There an Ideal Protocol?

Although many protocols with different doses and types of gonadotropins have been proposed in the literature over the past 20 years for the management of poor responder patients, to date there is no really efficient treatment that could solve the problem of poor ovarian response and the current question is still which is the ideal protocol for patients defined as “poor responders”?

### 5.1. Gonadotropins

When the standard dose of gonadotropins (225–300 IU) fails to induce a proper multifollicular growth, the obvious clinical approach is to increase the dose. High doses of gonadotropins have been thus used for a couple of decades by the vast majority of the authors in poor responder patients. In the literature conflicting data are however reported on the outcomes of this approach: some [38, 50, 51] but not all authors [52] in prospective and retrospective studies did not report enhanced ovarian response and/or better pregnancy rates when the starting dose of gonadotropins was increased up to 450 IU. More recently, Berkkanoglu and Ozgur [53] confirmed that the increase of FSH starting dose does not result in higher pregnancy rates and also found no differences between the starting dose of 300 UI, 450 UI, and 600 UI of gonadotropins in terms of retrieved oocytes, number of embryos obtained, and pregnancy rates. It is today clear that these patients have a reduced ovarian reserve; the recruitable follicles are fewer and the gonadotropins, independently of the dosage administered, can only support the cohort of follicles receptive to stimulation without manufacturing follicles de novo.

### 5.2. GnRH Analogues

From the beginning of the nineties the combination of gonadotropins and gonadotropin-releasing hormone (GnRH) agonists, started on the late luteal phase of the previous cycle, has been considered the protocol of choice in normoresponder patients. This approach lowers cancellation rate and raises the number of preovulatory follicles and the number of oocytes retrieved and good quality embryos for transfer, leading to better pregnancy rates [54]. However this protocol could have a detrimental effect in poor responders because it may induce an excessive ovarian suppression that could lead to a reduced or absent follicular response [55, 56]. For this reason, in patients with poor ovarian reserve the options could be

- to decrease the length of suppression by decreasing the duration of GnRH agonist use (short and ultrashort, mini- and microdose flareup regimens) [39, 44, 57],
- to lower or to stop (after pituitary suppression) the dose of GnRH agonists initiated during the luteal phase [41, 58],
- to use the GnRH antagonists in combination with gonadotropins to prevent premature LH rise during the mid-late follicular phase [59, 60].

Although short and ultrashort flareup regimens are widely used in poor responder patients for more than 20 years, all published studies, prospective nonrandomized trials with or without historical control [39, 44, 57], or retrospective ones [48, 61] were unable to demonstrate clearly any significant beneficial effect on the clinical outcome in this group of patients. Similarly, in a recent meta-analysis the clinical pregnancy rate per randomized patient and the duration of stimulation was not significantly different between patients treated by the short flareup regimens and the long GnRH agonist or GnRH antagonist protocol [62]. However, although with the short flareup protocols significantly more oocytes can be retrieved compared with GnRH antagonist protocols; the number of the retrieved oocytes is still significantly lower when compared with long standard GnRH regimens [62]. The rationale to lower or to stop (after pituitary suppression) the dose of GnRH agonists initiated during the luteal phase is to obtain a reduction of the inhibitory direct effect of the GnRH agonist on the gonads [55, 56]. Initially, several trials (prospective nonrandomized studies with historical control) on the use of “GnRH agonist stopped protocol” in poor responders reported a reduction in the amount of gonadotropin administered and improved laboratory results and clinical outcome [47, 63, 64]. Unfortunately, two prospective randomized controlled trials did not confirm improvements in reproductive outcome when this stimulation regimen is used compared to standard stimulation protocols [65, 66]. Similarly, in a recent meta-analysis no statistically significant difference was present in clinical pregnancy rates per cycle randomized between the “GnRH agonist stopped protocol” and the standard agonist protocol. Moreover, duration of stimulation and total number of gonadotropin ampoules required as well as number of oocytes retrieved were not statistically different between the two groups [62].

### 5.3. GnRH Antagonist

The use of GnRH antagonist was introduced in the clinical practice about 15 years ago. The most important advantages of the use of GnRH antagonist in combination with gonadotropins are improvement of patient's compliance, decreased number of days of stimulation and of the amount of gonadotropin administered, and statistically significant reduction of ovarian hyperstimulation syndrome (OHSS). Furthermore, GnRH antagonists are not administered during the stage of follicular recruitment and thus suppression of endogenous gonadotropins secretion is not present at that time in contrast to GnRH agonists being a possible advantage during ovarian stimulation in this group of patients. However, based on the last Cochrane database, it seems that use of GnRH antagonists, in the general population, may lead to a nonstatistically significant reduction of the live birth rates (OR 0.86, 95% CI 0.69 to 1.08) compared to GnRH agonist protocols, probably due to a different patient population selected for this stimulation regimen [67].

For these reasons, several authors suggested the use of GnRH antagonists in combination with gonadotropins as a suitable protocol for poor responders. In fact, GnRH antagonists in the mid-late follicular phase during ovarian stimulation prevent the premature LH surge while not causing suppression in the early follicular phase, obtaining more natural follicular recruitment without any inhibitory effect possibly induced by the GnRH agonist [68]. However, a meta-analysis of randomized controlled trials demonstrated that stimulation protocols where GnRH antagonist is used in combination with gonadotropins result in similar pregnancy rates compared with long agonist or short flareup regimens [69]. Similarly, in a more recent meta-analysis of 14 randomized controlled studies, GnRH antagonist protocols resulted in a statistically significant lower duration of stimulation compared with GnRH agonist protocols but there was no significant difference in the number of oocytes retrieved, in the cycle cancellation rate, and in the clinical pregnancy rate [70].

As there is not a stimulation protocol that significantly improves the clinical outcome in poor responder patients and that can be considered as a standard of medical care practice, the use of GnRH antagonist regimens could have some advantages over the GnRH agonist protocols. First, it is possible to assess the ovarian reserve by ultrasound on days 2-3 of the cycle in which controlled ovarian stimulation is planned and decide whether to initiate gonadotropins in the cycle where the probability of a favourable response is optimal. In fact, patients with a mean follicle count of <5 follicles have significant cycle-to-cycle variability in antral follicle count from −2 to +5 [71] to −3 to +7 [72]. Second, with use of GnRH antagonist to prevent premature LH rise we can utilize a new gonadotropin, a hybrid molecule with a prolonged half-life (corifollitropin alfa) that supports the cohort of follicle receptive to stimulation for seven days [73]. The use of these long acting gonadotropins could exploit fully the reduced ovarian reserve by the rapid increase in the serum FSH concentration that would result in a significantly higher exposure of the small antral follicles to constant high levels of FSH during the early follicular phase [74]. In a retrospective pilot study of poor responder patients according to the “Bologna criteria” Polyzos et al. [74] reported comparable laboratory and clinical results when corifollitropin alfa followed by rFSH in combination with GnRH antagonist was compared to conventional stimulation protocols. Finally, promising pregnancy rates are reported in a retrospective pilot study in young (<40 years) poor responder patients following a combination of corifollitropin alfa with hp-HMG in a GnRH antagonist protocol [75]. The peculiar pharmacokinetic of this molecule seems to be able to exploit fully the reduced ovarian reserve [73]. Moreover, the reduction of the number of daily subcutaneous injections of gonadotropins could reduce the physical and psychological burdens for these patients. These theories must encourage researchers to perform controlled randomized trials to validate this original ovarian stimulation regimen without significant risk of OHSS as observed in good prognosis patients [76].

## 6. Alternative Approaches

Several alternative approaches have been proposed with the aim of strengthening the effect of exogenous gonadotropins.

### 6.1. Addition of Estradiol in the Luteal Phase

In a recent meta-analysis of 8 selected studies from 1227 initially searched, the addition of estradiol in the luteal phase with or without the simultaneous use of GnRH antagonist decreases the risk of cycle cancellation and increases the chance of clinical pregnancy in poor responder patients [77]. The biological rationale might be that luteal estradiol priming could improve synchronization of the pool of follicles available to controlled ovarian stimulation [78]. However, this meta-analysis has been strongly criticized because of important methodological pitfall and randomized trials are needed before suggesting the addition of estradiol in the luteal phase, interpreting the results of this meta-analysis with caution [79].

### 6.2. Addition of Recombinant LH

Some authors suggested the addition of recombinant LH during gonadotropin stimulation in poor responder patients [80]. However, two meta-analyses [81, 82] showed that the addition of recombinant LH does not increase the number of oocyte retrieved, the total dose of FSH, the cancellation rates, and the ongoing pregnancy rates in poor responder patients. On the other hand, in a very recent meta-analysis of 40 randomized controlled studies [83], significantly more oocytes were retrieved and significantly higher clinical pregnancy rates were observed with r-hFSH plus r-hLH versus r-hFSH treatment in poor responders, suggesting that there is a relative increase in the clinical pregnancy rates of 30% in poor responders and that the addition of r-hLH to r-hFSH may be beneficial for women with poor ovarian response.

### 6.3. Addition of Growth Hormone

It has been suggested that the use of growth hormone (GH) might modulate the action of FSH on granulosa cells by upregulating the local synthesis of insulin-like growth factor-I (IGF-I) [84–86]. The IGF-I amplifies the effect of FSH at the level of both granulosa and theca cells [87, 88]. Two recent meta-analyses of 6 randomized trials (128 patients in total) [6, 62] suggested that addition of GH significantly increased the probability of live birth in poor responders. Regarding GH administration, frequency and dosage varied markedly among the eligible studies. However, due to the small number of the patients and the heterogeneity of the frequency and dosage of GH administered amongst the studies, the fact that the addition of GH during ovarian stimulation enhances the probability of pregnancy needs to be urgently evaluated in further properly designed trials to prove or disprove this finding. In fact, until now, there are not very recent and robust data suggesting routinary addition of GH in ovarian stimulation protocols for poor responders patients [89, 90].

### 6.4. Addition of Androgens

Androgens, produced primarily by theca cells, play a critical role for an adequate follicular steroidogenesis [91] and for a correct early follicular and granulosa cell development [92]. They are the substrate for the aromatase activity of the granulosa cells, which converts the androgens to estrogens. Moreover, androgens may increase FSH receptor expression in granulosa cells amplifying the effects of FSH and thus potentially enhance responsiveness of ovaries to FSH [6, 92, 93]. Furthermore, inadequate levels of endogenous androgens are associated with decreased ovarian sensitivity to FSH and low pregnancy rates after IVF [6, 94].

Based on these observations Casson et al. [95] first suggested that the oral administration of dehydroepiandrosterone (DHEA) before ovarian stimulation with gonadotropin could improve the response in poor responder patients. In the last decade several uncontrolled studies published improved clinical outcome after the oral administration of DHEA before ovarian stimulation. A recent meta-analysis of four randomized controlled trials of adjuvant androgens (DHEA and testosterone) in poor responder patients showed a significantly higher ongoing pregnancy rate in the androgen supplementation group [96]. However, the included studies were too small and presented clinical and methodological heterogeneity to be conclusive and to warrant an immediate change in practice. Actually, there is a need of robust data from randomized controlled studies that could justify the widespread use of DHEA before ovarian stimulation in poor responders and it is time to evaluate the clinical cost effectiveness of DHEA with large multicentre randomized controlled trials [97].

### 6.5. Addition of Aspirin

Increased intraovarian vascularity has been linked to improved delivery of gonadotropic hormones or other growth factors required for folliculogenesis [98, 99]. On the other hand, impaired ovarian blood flow could contribute to poor ovarian response [100, 101]. Based on this rationale, by enhancing ovarian vascularization with vasoactive substances such as aspirin, the ovarian response could theoretically improve [102].

However, the evidence supporting the effect of a low dose of aspirin in women undergoing IVF is poor and controversial [103]. Although some papers have reported some beneficial effects of aspirin from the day of embryo transfer [102, 104] others have failed to confirm these findings, also in poor responders [105–107]. Prospective randomized trials demonstrated that adjuvant therapy with aspirin and prednisolone did not improve uterine blood flow, implantation, and pregnancy rates [108]. The conclusion of a meta-analysis and a systematic review [109] was that clinical pregnancy rate per embryo transfer was not found to be different between patients who received low-dose aspirin and the control group. On the basis of updated evidence, a low dose of aspirin has no substantial positive effect on the likelihood of pregnancy and it should not be routinely recommended for women undergoing IVF.

### 6.6. Natural Cycles IVF

Natural cycles IVF with or without minimal stimulation can be considered as an easy and cheap approach in the management of poor responders. In fact, some authors suggested that natural cycles IVF was a valid option for poor responder patients because it has the same chance in terms of pregnancy and implantation rates [110]. Some critical issues have been highlighted: in only 50% of started cycles one embryo could be replaced into the uterine cavity (50% cancellation rate); the overall clinical pregnancy rate was 10% per started cycle and 18% per patient and per embryo transfer [111]. Schimberni et al. [112] evaluated the IVF outcome in a large group of poor responders (500 patients) reporting very encouraging results especially in younger woman (<35 years). In this group of poor responder patients the pregnancy rate was 18% per started cycle, 29% per transfer, and 31% per patient. In contrast, a recent paper analyzed the effect of natural cycles IVF in women defined as poor responders according to the “Bologna criteria”: unexpectedly the data showed that the cumulative live birth per patient does not exceed 8% [113]. The contradictory data can be very likely associated to the diversity of patients selected before the application of “Bologna criteria.”

### 6.7. Oocyte Cryopreservation

Oocyte vitrification has resulted in the breakthrough in ART technologies, probably the most important one in our decade. Recent evidence indicates that this approach is a highly efficient procedure that can be applied in routine infertility programs [114]. The major societies including European Society of Human Reproduction and Embryology (ESHRE), American Society for Reproductive Medicine (ASRM), and American Society of Clinical Oncologists (ASCO) acknowledged recently oocyte cryopreservation as a nonexperimental procedure which provides the required legal and moral support for widespread application [115]. Different strategies are applicable for poor responder involving oocyte cryopreservation. Some authors have recently suggested obtaining a large cohort of oocytes in these patients by accumulating vitrified oocytes over several stimulation cycles creating a similar situation as in normal responder patients. According to the results presented in the study, it could be possible to obtain higher live birth rate per patient treated and potentially to reduce the dropout [116]. Moreover, oocyte cryopreservation can also be used to preserve the fertility of all those women at risk to lose their ovarian potential over the time.

## 7. Conclusions

Despite the huge number of papers that have been published in the last two decades trying to find most efficient stimulation protocol for the management of patients with poor ovarian response, at the moment systematic reviews and meta-analyses suggest that insufficient evidence exists to recommend most of the treatments proposed to improve pregnancy rates, with the poor ovarian response remaining one of the most challenging tasks in reproductive medicine. One may speculate that the effect of any medication suggested for this group of patients is insignificant or even negligible. By reviewing the applied treatment, we only found a single exception. A very promising stimulation protocol is in fact represented by the combination of a long acting gonadotropin (corifollitropin alfa) with HP-HMG in a GnRH antagonist regimen. However, prospective studies are necessary to confirm the possible benefit of this approach.

To properly design studies able to clarify which is the best approach for this group of patients, a homogeneous population must be included based on a clear definition of poor response. The work performed by the panel of experts in reproductive medicine gathered together in the ESHRE Campus in Bologna in 2011 is highly appreciated. This can be considered an important step forward to the identification of poor responder patients and consequently to find the most effective strategy in their management. Finally, we should think also about alternative strategies able to prevent the consequences of the poor ovarian response. Oocyte cryopreservation for fertility preservation before the ovarian reserve decline is an important option that must be offered. As available evidence does not indicate any harmful consequence of oocyte vitrification [117, 118] it is not just a possibility but a moral duty to exploit fully its potentials in various situations including the prevention of poor ovarian response.
